# Targeting Apoptotic Pathways in Acute Myeloid Leukaemia

**DOI:** 10.3390/cancers11111660

**Published:** 2019-10-26

**Authors:** Jonathan R. Sillar, Anoop K. Enjeti

**Affiliations:** 1 School of Biomedical Sciences and Pharmacy, The University of Newcastle, Callaghan, NSW 2308, Australia; Jonathan.Sillar@health.nsw.gov.au; 2 Haematology Department, Calvary Mater Hospital Newcastle, Waratah, NSW 2298, Australia; 3 NSW Health Pathology North- Hunter John Hunter Hospital, Newcastle, NSW 2305, Australia; 4 School of Medicine and Public Health, The University of Newcastle, Callaghan, NSW 2308, Australia

**Keywords:** Acute Myeloid Leukaemia, programmed cell death, BCL-2, MCL-1, BH3 mimetic, apoptosis

## Abstract

Acute Myeloid Leukaemia is a devastating disease that continues to have a poor outcome for the majority of patients. In recent years, however, a number of drugs have received FDA approval, following on from successful clinical trial results. This parallels the characterization of the molecular landscape of Acute Myeloid Leukaemia (AML) over the last decade, which has led to the development of drugs targeting newly identified recurring mutations. In addition, basic biological research into the pathobiology of AML has identified aberrant programmed cell death pathways in AML. Following on from successful outcomes in lymphoid malignancies, drugs targeting the B Cell Lymphoma 2 (BCL-2) family of anti-apoptotic proteins have been explored in AML. In this review, we will outline the preclinical and clinical work to date supporting the role of drugs targeting BCL-2, with Venetoclax being the most advanced to date. We will also highlight rationale combinations using Venetoclax, ongoing clinical trials and biomarkers of response identified from the early phase clinical trials performed.

## 1. Introduction

Acute Myeloid Leukaemia (AML) is characterized by the clonal proliferation of immature cells of myeloid lineage that leads to bone marrow failure and death without treatment. According to the Australian Institute of Health and Welfare, there are approximately 1000 new cases of AML per annum with 900 deaths, with a 5 year overall survival of only 24.5% [1]. In older patients, hypomethylating agents, low dose cytarabine or supportive care is the standard of care with a less than 10% overall survival [2]. In younger patients, the “7+3” induction chemotherapy regimen, followed by consolidation with an allogeneic stem cell transplant, remains the best chance for a cure, apart from the favourable risk group, in whom consolidation with high dose cytarabine is curative in the majority of patients who achieve complete remission (CR) after induction. In recent years, the molecular landscape of AML has been characterized in great detail [3,4]. This is reflected in the most recent edition of the World Health Organization classification of myeloid neoplasms and acute leukemia, which includes a number of new diagnostic categories of AML defined by their molecular, rather than morphological, features [5]. Further, the European Leukaemia Net 2017 risk stratification of AML includes recurring mutations in six genes (*FLT3*, *CEBPA*, *NPM1*, *RUNX1*, *ASXL1* and *TP53*), and the well-characterized cytogenetic changes [6]. In addition, this has afforded a greater ability to identify targetable mutations, such as those recurring in the Fms-like tyrosine kinase 3 (FLT3) and isocitrate dehydrogenase 1 and 2 (IDH1/2). Indeed, we have now seen FDA approvals for drugs targeting FLT3 (midostaurin, gilteritinib), IDH1 (ivosidenib) and IDH2 (enasidenib) in recent years based on the results of Phase 2 and 3 clinical trials for relapsed disease [7,8,9,10]. Further, drugs agnostic to the underlying genetic landscape have seen approval, including the antibody drug conjugate gemtuzumab ozogamicin, which targets CD33; the hedgehog pathway inhibitor glasdegib; and the B cell lymphoma 2 (BCL-2) inhibitor venetoclax, with promising results. This review will outline the role of apoptotic pathways in promoting leukaemogenesis in AML, the development of BCL-2 inhibitors over time that has led to the recent approval of venetoclax by the FDA and ongoing trials targeting dysregulated apoptotic pathways in AML.

## 2. Apoptosis and the BCL-2 Family of Proteins

Apoptosis is a form of programmed cell death that, via a cascade of highly regulated processes, maintains cellular homeostasis. There are two predominant pathways that are thought to initiate apoptosis; namely, the intrinsic and extrinsic pathways [11]. The extrinsic pathway is initiated via external stimuli activating tumour necrosis factor (TNF) family receptors, that, in turn, activate downstream adaptor proteins, including caspases, which eventually leads to cell death via apoptosis [12]. The intrinsic pathway is triggered by internal cellular stress, such as DNA damage. Caspase activation is linked to mitochondrial outer membrane permeabilisation (MOMP), which, in turn, is regulated by the B cell Lymphoma 2 (BCL-2) family of proteins [13]. This review will focus on novel agents that have been designed to target the intrinsic pathway of apoptosis.

BCL-2 was first identified as a pro-survival oncogene in Follicular Lymphoma, where the characteristic translocation, t(14;18), couples the immunoglobulin heavy chain promotor with the *BCL-2* gene [14]. It was soon established that BCL-2 promotes lymphomagenesis through blocking programmed cell death [15,16]. Since the discovery of BCL-2, five other anti-apoptotic proteins in the BCL-2 family have been identified: BCL-xL, MCL-1, BCL-W, BFL-1 and BCL-B [17]. These anti-apoptotic proteins contain four BH domains, named BH1–BH4. Overexpression of these proteins has been well characterized in both haematological and non-haematological malignancies alike [18]. Following on from the identification of BCL-2, attempts were made to discover pro-apoptotic proteins. The BCL-2 associated protein X (BAX), was the first identified by Oltvai et al. in 1993, and was found to have a surprising homology to BCL-2 [19]. A vast number of pro-apoptotic proteins in the BCL-2 family have now been identified and their complex interactions, with the anti-apoptotic proteins regulating cell survival having been characterized (reviewed in [20]). In brief, pro-apoptotic proteins can be divided into effector proteins (BAX, BAK and BOK), which contain three to four BH domains and the BH3-only proteins (BID, BIM, BAD, BIK, NOXA, PUMA, HRK and BMF). The effector proteins, once activated, oligomerize and form pores in the outer mitochondrial membrane leading to MOMP, cytochrome c release and ultimately cell death [21,22]. The BH3-only proteins can be further subdivided into activator and sensitizer proteins. The activator BH3-only proteins, namely BID and BIM, lead to oligomerization of the activator proteins upon direct binding, whereas the sensitizer proteins exert their pro-apoptotic effect by displacing the activator proteins from the BH3 binding pockets in the anti-apoptotic BCL-2 proteins [20]. This detailed understanding and the demonstrated role of BCL-2 family proteins in promoting tumorigenesis has led to a number of novel drugs targeting aberrant apoptotic pathways in cancer.

In the case of AML, a number of papers were published in the early 1990s implicating BCL-2 in promoting leukaemogenesis and conferring resistance to treatment [23,24,25]. This led to the early development of antisense oligonucleotides targeting BCL-2, that were shown to slow leukaemic cell growth and survival in vitro [26]. Later work implicated other BCL-2 family proteins, including BCL-xL and MCL-1, in the pathogenesis of AML [27,28,29]. With a greater understanding of the intrinsic apoptotic pathway came the development of BH3 mimetic drugs. These novel agents mimic the action of certain pro-apoptotic BH3-only proteins, and thus induce apoptosis. Following on with the successful results in lymphoid neoplasms, BH3 mimetics have been utilized in AML, with significant promise.

## 3. Targeting BCL-2 in AML

### 3.1. Oblimersen (BCL-2 Anti-Sense Oligonucleotide)

Oblimersen is an anti-sense oligonucleotide and was the first drug targeting BCL-2 used in clinical trials of AML. The drug, which is prepared as an intravenous formulation, targets the first six codons of the human BCL-2 mRNA, leading to decreased protein expression. In preclinical leukaemia models, this had been shown to lead to increased apoptosis [26,30]. Phase 1 trials in lymphoma and prostate cancer demonstrated tolerability of the drug in humans with clinical responses observed [31,32]. This led to a Phase 1 trial in relapsed and refractory patients with acute myeloid and acute lymphoblastic leukaemia, in combination with fludarabine, cytarabine and granulocyte colony-stimulating factor (FLAG) salvage chemotherapy [33]. Overall, twenty patients were recruited, of which seventeen patients had AML. Complete responses (CRs) were observed in five of 17 (29%) AML patients, with a further two patients (12%) achieving CRs with incomplete haematological responses (CRis). Of note, no dose limiting toxicities were observed. Another Phase 1 trial performed by the same group combined oblimersen with chemotherapy in untreated older patients with AML [34]. Fourteen of 29 patients (48%) achieved a CR, with a decrease in BCL-2 mRNA copies observed in responders. Interestingly non-responders showed an increase in BCL-2 mRNA copies. Again, the drug was considered tolerable compared to standard induction therapy. Following on from these Phase 1 trials, the CALGB performed a Phase 3 randomised trial in previously untreated patients with AML, older than 60 years of age [35]. Patients were treated with the standard “7+3” induction chemotherapy regimen followed by high dose cytarabine (HiDAC) consolidation with (Arm A) or without (Arm B) oblimersen. Unfortunately, there was no difference observed in CR (48% versus 52%; *p* = 0.75) or in overall survival, which ultimately led to no further trials of this drug in AML.

### 3.2. Obatoclax

Obatoclax was the first BH3 mimetic to move forward into clinical trials for AML. In pre-clinical models, it was shown to inhibit BCL-2 and related family members BCL-xL, MCL-1, BCL-w, A1 and BCL-b [36]. In AML cell lines and primary AML samples, obatoclax was shown to induce apoptosis and slow leukaemic cell proliferation [37]. In addition, responses and safety were demonstrated in vivo using a mouse xenograft model of Myeloma and solid tumour cell lines [36,38]. Obatoclax was initially tested in a Phase 1 trial of 44 patients with advanced haematological malignancies, including subjects with refractory AML (57%), myelodysplasia (MDS) (32%), chronic lymphocytic leukaemia (CLL) (9%) and acute lymphoblastic leukaemia (ALL) (2%) [39]. The drug appeared well tolerated, with some grade 1/2 central nervous system side effects being the most common adverse events. The responses, however, were modest. Only one case of refractory AML with a t(9;11) translocation went into a CR, and only three out 14 patients with myelodysplasia showed haematological improvement. In other Phase 1 trials of single-agent obatoclax, responses were seen in CLL, Hodgkin’s and non-Hodgkin’s Lymphoma, albeit modest [40,41,42].

A follow up Phase 1/2 study using obatoclax in older patients (>70 years) with untreated AML was performed based on the tolerable safety profile in the previous trial [43]. The Phase 2-component randomised patients used two different preparations of obatoclax to attempt to identify the optimal formulation of the drug. Nineteen patients were recruited with a median age of 81 years. Unfortunately, no patients demonstrated a complete response and only four patients showed stable disease. The drug was, again, well tolerated apart from mild neurological and cardiac events; however, based on the disappointing responses, the drug was not further developed in AML.

### 3.3. ABT-737/ABT-263 (Navitoclax)

ABT-737 was developed by Abott Laboratories (now AbbVie) as a BCL-2 family inhibitor using nuclear magnetic resonance (NMR)-based screening [44]. It was shown to inhibit BCL-2, BCL-xL and BCL-w with a high potency. Apoptosis upon treatment with ABT-737 in vitro was demonstrated to be BAX/BAK dependent, as opposed to a number of other BH3 mimetics available at the time, and suggested an “on-target” mechanism of action [45]. In AML cell lines and primary AML samples, ABT-737 inhibited cell growth in vitro, and a murine xenograft model demonstrated activity in vivo [29]. High MCL-1 expression was shown to confer resistance to ABT-737, with restoration of drug activity upon MCL-1 knockdown using shRNA [29,45]. Similarly, by combining ABT-737 with a MEK inhibitor in vitro and in vivo, synergistic cytotoxicity was demonstrated by inhibiting mitogen activated protein kinase (MAPK) dependent MCL-1 expression [46]. Further, synergistic apoptosis was observed in AML cell lines and primary AML samples with activating FLT3 mutations after combining ABT-737 with two different FLT3 inhibitors [47]. Despite this promising pre-clinical data for ABT-737 in AML, its lack of oral bioavailability and water insolubility hampered translation of this drug into clinical practice. This led to the development of ABT-263 as a novel BH3 mimetic. ABT-263 (navitoclax) is an orally-bioavailable BH3 mimetic with high affinity binding for BCL-2, BCL-xL and BCL-w that disrupts protein–protein interactions between BCL-2/BCL-xL and pro-apoptotic proteins [48]. As with ABT-737, ABT-263 demonstrated pre-clinical efficacy in AML models [49,50,51,52]. ABT-263 has been tested in clinical trials for patients with CLL and solid organ tumours with a consistent dose, limiting the toxicity of thrombocytopenia due to its effect on BCL-xL [53,54]. To date, no clinical trials of ABT-263 have been undertaken in AML, likely in part due to this ‘on target’ effect and the development of ABT-199.

### 3.4. Venetoclax (ABT-199)

Venetoclax (formerly ABT-199) is a BCL-2 specific BH3 mimetic. It was cleverly reverse-engineered using the crystal structure of Navitoclax to identify the BCL-xL and BCl-2 binding sites, with modifications made to create a molecule with selective binding to BCL-2 [55]. Venetoclax was shown to have sub-nanomolar affinity for BCL-2 compared with nanomolar affinity for BCL-xL and BCL-w. This correlated with the sparing of platelets treated ex vivo compared with Navitoclax, and no significant thrombocytopenia was observed in dogs in vivo at doses considered therapeutic based on preclinical models of haematological malignancies. The authors of this seminal paper went on to assess the safety and efficacy of venetoclax in patients with refractory CLL. A dramatic response was observed with tumour lysis seen after administration of a single dose of venetoclax in the first three patients exposed. Subsequent large Phase 3 trials have established venetoclax as a potent agent in the treatment of CLL in both upfront and relapsed/refractory settings [56,57]. In AML, venetoclax was quickly established as a promising agent in pre-clinical studies. The potency of venetoclax was demonstrated in AML cell lines in vitro, in primary AML samples treated ex vivo and using a murine xenograft model in vivo [58,59]. Venetoclax was quickly moved into clinical trials of AML based on this work, along with the ABT-767 and Navitoclax data. In addition, safety in humans had already been demonstrated in CLL, allowing rapid translation to the clinic.

#### 3.4.1. Venetoclax Monotherapy in Relapsed/Refractory Patients

The first trial of venetoclax in AML recruited 32 patients (median age 71 years) with either relapsed/refractory disease or untreated patients unfit for intensive chemotherapy [60]. All but two patients, however, had received one or more lines of prior therapy. Indeed, thirteen patients (41%) had received three or more prior treatments consistent with a heavily pre-treated cohort. Further, 41% of patients had a prior MDS or myeloproliferative neoplasm (MPN), and 31% had a complex karyotype. Treatment consisted of single-agent venetoclax, with a ramp-up schedule to mitigate the risk of tumour lysis syndrome (TLS) and a target dose of 800 mg. Escalation to 1200 mg was allowed for patients not achieving CR or CRi at 4 weeks and who were tolerant to the drug. The overall response rate was 19%, with 6% achieving a CR and 13% a Cri, and responses, for all but one patient, occurred within 4 weeks. Higher responses were observed in the IDH1/2 mutant patients with 33% of patients achieving a CR/CRi. This is consistent with preclinical studies in which IDH1/2 mutant AML, via the oncometabolite (R)-2-HG, inhibits the activity of cytochrome c oxidase (COX) in the mitochondrial electron transport chain (ETC), which in turn, lowered the mitochondrial threshold to trigger apoptosis upon BCL-2 inhibition [61]. The median duration of CR was only 48 days and the median time spent on the study was only 63.5 days. In general, venetoclax was well tolerated, with the most common grade 3/4 adverse events (AEs) being febrile neutropenia, hypokalaemia, pneumonia, hypotension and urinary tract infections, all of which would be expected in this cohort of patients [62,63]. There were no reported episodes of TLS. In summary, single-agent venetoclax was demonstrated to be tolerable, with response rates being promising for such a heavily pre-treated cohort of patients.

#### 3.4.2. Venetoclax + Hypomethylating Agents in Treatment-Naïve Patients

Venetoclax was subsequently examined in combination with hypomethylating agents (HMAs), both azacitidine and decitabine. Pre-clinical in vitro studies had suggested a synergistic effect upon combining BH3 mimetics and HMAs in AML cell lines and primary AML patient samples [64]. Further, azacitidine had been shown to reduce MCL-1 levels, another anti-apoptotic protein not targeted by venetoclax and a potential source of venetoclax resistance [65,66]. A phase 1b/2 dose escalation and expansion study was conducted and included 145 patients with treatment-naïve AML over the age of 65 years (median age 74 years). In the dose escalation phase, venetoclax at 400, 800 and 1200 mg once daily doses was administered in combination with either azacitidine (75 mg/m^2^, days 1–7, intravenously or subcutaneously) or decitabine (20 mg/m^2^, days 1–5, intravenously) in 28-day cycles. In the expansion phases, doses of 400 and 800 mg of venetoclax were chosen based on the preliminary safety and efficacy data [67]. A ramp-up dosing schedule of venetoclax was used and all patients hospitalised for at least 3 days. Using this schedule, no clinical tumour lysis syndrome was observed. The reasons for less far TLS in AML compared with CLL is not known apart from the slow ramp-up and TLS precautions that were used. Approximately equal numbers of patients received either azacitidine or decitabine. Patients who had previously received HMAs or chemotherapy were excluded, as were patients with favourable risk cytogenetics. Fifty-one percent had intermediate-risk cytogenetics, 49% had poor-risk cytogenetics and 25% had secondary AML. The overall response rates were remarkable at 68% and included CR and CRi rates of 37% and 30% respectively. The median time to the first response was only 1.2 months, compared to approximately 4 months for HMAs alone [68,69]. The median overall survival was 15.1 months, which compares favourably to the 10.4 months seen with azacitidine alone in the Phase 3 AZA-AML-001 study, albeit with the standard caveats of cross-trial comparisons [70]. Subgroup analyses showed high response rates in patients with mutations in IDH1/2 (71% CR/CRi), NPM1 (91.5% CR/CRi) and FLT3 (72% CR/CRi), and those with intermediate cytogenetics (74% CR/CRi). Even patients with adverse risk features showed impressive responses, including those with poor risk cytogenetics (60% CR/CRi) and TP53 mutant patients (47% CR/CRi). Adverse events were primarily haematological and expected for this cohort of patients. No significant difference was observed between the azacitidine and the decitabine groups and no difference was observed between the 400 and 800 mg venetoclax groups. The promising responses observed in this trial led to the FDA approval of venetoclax in combination with HMAs for the treatment of unfit, elderly patients with AML in November 2018. An ongoing Phase 3 trial comparing venetoclax (400 mg) + azacitidine with azacitidine alone has completed accrual, with follow up ongoing and the results being eagerly awaited (NCT02993523).

#### 3.4.3. Venetoclax + Low Dose Cytarabine in Treatment-Naïve Patients

In addition to HMAs, venetoclax has been tested in combination with low dose cytarabine (LDAC) in an upfront setting. The rationale for this combination is supported by pre-clinical studies demonstrating venetoclax/LDAC synergy and reduced MCL-1 protein levels with combination therapy compared to venetoclax alone [71,72]. An international phase 1b/2 study recruited 82 patients with treatment-naïve AML over the age of 60 years (median age 74 years). In total, 49% had secondary AML, 29% had prior HMA treatment for MDS and 32% had poor-risk cytogenetic features. In the dose escalation phase, higher rates of haematological toxicity were observed with the 800 mg dose of venetoclax, and therefore, a 600 mg dose was chosen for the Phase 2 component of the trial in which patients received venetoclax 600 mg daily with LDAC (20 mg/m^2^, days 1–10, subcutaneously) administered in 28-day cycles. The CR/CRi rate was 54% with a median time to response of 1.4 months. Excluding patients with prior HMA exposure, the CR/CRi rate was 62%, comparable to the 67% observed with venetoclax + HMAs. The median overall survival was 10.1 months for all patients and 13.5 months for patients without prior HMA exposure. Subgroup analyses again, showed better than average responses in patients with mutations in NPM1 or IDH1/2 mutations with CR/CRi rates of 89% and 72% respectively. Patients with TP53 or FLT3 mutations had worse CR/CRi rates (30% and 44%), notably lower than in the venetoclax + HMA trial discussed above. Toxicities were primarily haematological, as expected. Laboratory TLS was observed in two patients with no evidence of clinical TLS likely, owing to the slow ramp-up schedule and the use of prophylactic medications. In summary, these results are again, very promising and far improved over historical controls of LDAC alone (CR/CRi rate of 19%) [73]. The combination of venetoclax 600 mg and LDAC is also being tested in a Phase 3 clinical trial compared to LDAC alone, with accrual finished and follow up ongoing (NCT03069352).

#### 3.4.4. Venetoclax + HMA/LDAC in Relapsed/Refractory Patients

Although not directly tested in a clinical trial, retrospective studies of relapsed or refractory patients receiving venetoclax plus HMAs or LDAC have been reported. In a series of 33 patients post either HMAs (61%) or allogeneic stem cell transplants (39%), the combination of venetoclax and either azacitidine or decitabine achieved a CR/CRi rate of 33% [74]. In another series of 24 patients, treated with the combination of venetoclax and HMA (*n* = 8) or venetoclax and LDAC (*n* = 16), the composite CR rate was 24% [75]. Whilst in yet another series of 43 patients with relapsed/refractory myeloid neoplasms of which 91% were AML cases, the CR/CRi rate was a dismal 12%, with a median overall survival of 3 months [76]. This is comparable to the 19% CR/CRi observed with single-agent venetoclax [60]. Hence, with the data available, the value of venetoclax as a salvage therapy either alone or in combination with HMAs/LDAC appears comparable with standard salvage regimens [77].

### 3.5. Ongoing Clinical Trials Using Venetoclax in AML

#### 3.5.1. Phase III Trials of Venetoclax + Azacitdine/LDAC in Treatment-Naïve Patients

Given the promising results described above, there is a huge amount of interest in combining venetoclax with standard backbone chemotherapeutics and novel targeted agents with a number of early phase trials actively recruiting (Table 1). The vast majority of these trials are early phase with few patients recruited to date. The only Phase 3 trials active have already been mentioned and include venetoclax and either azacitidine or LDAC versus azacitidine or LDAC alone. If these trials result in positive outcomes, venetoclax will be established as the standard of care in the upfront treatment or elderly patients with AML, however, until these results are published access to venetoclax is restricted to clinical trials in many countries outside of the United States.

#### 3.5.2. Venetoclax + Intensive Chemotherapy

As expected, trials are currently underway combining venetoclax with chemotherapeutic regimens, including the FLAG-IDA (fludarabine, cytarabine, filgrastim, idarubicin) salvage regimen (NCT03214562), “7+3” induction chemotherapy (NCT03709758) and the novel CPX-351 (NCT03629171). At the time of writing, preliminary data was only available for the FLAG-IDA combination (known as FLAG-V-I) [78]. The trial is recruiting relapsed/refractory patients over the age of 18. In an interim analysis of 11 patients with response assessments available, eight patients (73%) achieved a CR/CRi. The safety profile was acceptable with no early mortality and severe adverse events expected for such an intensive regimen. The median time to neutrophil recovery was 28 days, which again, is not unexpected for FLAG-IDA. Preliminary results of a phase 1b study in elderly patients (age > 65) combining venetoclax with a modified cytarabine and idarubicin induction and consolidation backbone was reported at the 2018 ASH meeting [79]. Patients received 14 days of venetoclax with each cycle of chemotherapy, followed by seven cycles of venetoclax monotherapy as maintenance. The overall CR/CRi was 71%, with an impressive 95% observed in de novo AML cases. The best responses were observed in patients with NPM1 (100%), RUNX1 (90%), IDH1/2 (89%) and RAS (90%) mutations, while those with TP53 (33%) mutations fared the worst. Remarkably, NPM1 minimal residual disease (MRD) negativity was demonstrated in 83% of patients with NPM1 mutations. The question remains as to whether such high-intensity treatment is required, especially given the high responses observed by combining venetoclax with lower-intensity therapies, such as HMAs and LDAC.

#### 3.5.3. Venetoclax + FLT3 Inhibitors

Venetoclax is also being explored in combination with FLT3 inhibitors. Current ongoing trials include a Phase 1 study combining venetoclax with gilteritinib in relapsed/refractory FLT3 mutant AML patients (NCT03625505) and a Phase 1/2 trial combining venetoclax with quizartinib in a similar cohort (NCT03735875). There are pre-clinical models supporting this as a rational combination. Notably, a synergistic effect has been observed in AML cell lines and primary AML blasts treated in vitro with the combination of ABT-737 and the FLT3 inhibitors, sunitinib and SU5614 [47]. In addition, a recent paper highlighted a synergistic effect of venetoclax combined with midostaurin or gilteritinib in vivo using a murine FLT3-ITD AML cell line-derived xenograft model [80]. Midostaurin and gilteritinib were shown to downregulate MCL-1 expression, which may, in part, explain the synergistic cytotoxicity observed. The combination of quizartinib and venetoclax has also been explored with increased survival observed using the combination in a murine FLT3-ITD AML model [81]. The authors demonstrated reduced MCL-1 and BCL-xL, but not BCL-2 expression in FLT3-ITD+ cell lines upon treatment with quizartinib, confirming the FLT3-ITD mediated regulation of MCL-1 and BCL-xL. In summary, the combination of venetoclax and FLT3 inhibitors is in early development, with preliminary safety data being awaited. Given the ongoing adverse prognostic implication of FLT3 mutations in the HMA/LDAC + venetoclax combinations, it will be interesting to see if exchanging an HMA/LDAC for a FLT3 inhibitor will result in improved responses.

#### 3.5.4. Venetoclax + IDH1/2 Inhibitors

The combination of venetoclax + ivosidenib (IDH1 inhibitor), with or without azacitdine is being explored in a Phase 1b/2 trial of patients with relapsed/refractory AML or treatment-naïve patients not fit for intensive chemotherapy (NCT03471260). There does not appear to be any current clinical trials combining the IDH2 inhibitor, enasidenib, with venetoclax at this point in time. Both ivosidenib and enasidenib have been approved by the FDA for use in patients with relapsed/refractory AML with IDH1 and IDH2 mutations respectively. BCL-2 dependence has been demonstrated in primary AML blasts harbouring IDH mutations, and improved anti-leukaemic activity was observed in a murine IDH2 mutant model of AML by combining venetoclax with enasidenib [61,82]. As discussed earlier, the response rates of IDH1/2 mutant patients treated with venetoclax + HMAs/LDAC was ~70% suggesting this subset of patients is particularly vulnerable to BCL-2 inhibition.

#### 3.5.5. Venetoclax + JAK Inhibitors (Ruxolitinib)

The evidence supporting this combination comes from a paper that studied the ex vivo responses of primary AML blasts in bone marrow stroma-derived and standard culture conditions when exposed to various approved and investigational agents [83]. A number of agents, including venetoclax, displayed reduced sensitivity when tested on patient samples in BM stroma-derived co-cultures compared to standard culture conditions. The JAK inhibitors displayed the reverse pattern. Interestingly, ruxolitinib was able to restore venetoclax sensitivity in AML patient cells tested in ex vivo models and in vivo using an AML xenograft mouse model. Mechanistically, the bone marrow stroma confers venetoclax resistance by reducing the primary AML cells’ BCL-2 dependency with decreased BCL-2 expression and increased BCL-xL and BCL-xS expression. The upstream effectors of this appear to be G-CSF and GM-CSF secreted from the stromal cells, which leads to increased phosphorylation of STAT5, a downstream effector of JAKs. Hence, JAK inhibition with ruxolitinib maintains BCL-2 dependency in the AML blasts through suppression of the JAK-STAT pathway. Based on this pre-clinical work, a Phase 1 trial is exploring this combination in a relapsed/refractory population (NCT03874052).

#### 3.5.6. Venetoclax + MCL-1 Inhibitors

Venetoclax is being trialled in combination with novel MCL-1 inhibitors in relapsed/refractory patients, S64315 (NCT03672695) and AMG 176 (NCT03797261). It is also being trialled with agents that have been shown to reduce MCL-1 expression levels, including the MEK inhibitor, cobimetinib (NCT02670044), and the cyclin-dependent kinase inhibitors, dinaciclib (NCT03484520) and alvocidib (NCT03441555). Given its selectivity for BCL-2, an intrinsic mechanism of venetoclax resistance is due to increased AML blast dependency on the anti-apoptotic proteins, BCL-xL and MCL-1. In the Phase 2 trial of venetoclax monotherapy in relapsed/refractory AML patients, both BCL-xL and MCL-1 expression levels negatively correlated with response to venetoclax [60]. A numbers of novel therapies tested in pre-clinical models in combination with venetoclax have demonstrated synergistic effects by downregulating MCL-1 expression [46,49,84,85]. Indeed, azacitidine has also been shown to reduce MCL-1 expression, as previously mentioned [65]. Hence, directly targeting MCL-1 makes logical sense in combination with venetoclax in AML. As proof of concept, a recent study investigated the role of BCL-2 and MCL-1 in AML survival by combining inducible lentiviral vectors expressing BH3-only proteins [71]. Targeting BCL-2 and MCL-1 improved survival in a mouse xenograft model, whereas other combinations including BCL-2/BCL-XL/BCL-W (akin to ABT-737) or MCL-1 alone, did not. Hence, combining venetoclax with an MCL-1 inhibitor is an exciting prospect for the treatment of patients with AML. Preliminary results from the Phase 1b trial combining cobimetinib and venetoclax show overall responses of 18% in a heavily pre-treated population, with gastrointestinal toxicity being the major adverse toxicity [86]. There are no preliminary results from the combination of the direct MCL-1 inhibitors, S64315 and AMG 176, and venetoclax, to date.

#### 3.5.7. Venetoclax + MDM2 Inhibitors

Idasanutlin is a novel MDM2 inhibitor that is being tested in combination with venetoclax (NCT02670044). MDM2 is a negative regulator of wild-type p53 (WT-p53). In AML, TP53 mutations occurs in only 7–8% of de novo cases, whereas inactivation of WT-p53 occurs in almost all subsets, making disruption of the MDM2 and WT-p53 interaction a promising target [87]. Pre-clinical models have shown that approximately two thirds of AML cell lines and primary AML blasts respond to MDM2 inhibition, with resistance observed in the TP53 mutant samples, as expected [88,89]. The combination of venetoclax and MDM2 has been tested using in vitro and in vivo models of AML, with synergistic responses having been observed [85,90]. Preliminary results from the Phase 1b trial combining venetoclax with idasanutlin demonstrated a 38% overall response in the higher dose cohort, with no responses in the TP53 mutant patients [86].

### 3.6. Predictors of Response to Venetoclax

#### 3.6.1. Molecular Subtypes of AML

The data from the completed early phase clinical using venetoclax in patients with AML highlights differential responses across cytogenetic and molecularly defined AML subgroups (Table 2). In the upfront setting, patients with NPM1 and IDH1/2 have the most favourable responses with the CR/CRi rates reported as ~90% and ~70% respectively, when combined with either HMAs or LDAC. Patients with TP53 mutations had CR/CRi rates of 44% when combined with HMAs, and only 30% when combined with LDAC. Those patients with FLT3 mutations had responses of 72% when combined with HMAs and 44% when combined with LDAC. Patients with poor risk cytogenetics demonstrated inferior response rates to those with intermediate risk profiles as expected. Patients with favourable risk cytogenetics, notably the core binding factor AMLs, were excluded from the trials, so no response data is available for that group to date. As discussed earlier, preliminary results combining venetoclax with cytarabine/idarubicin in elderly patients with AML, show deep responses in NPM1 mutated cases (83% MRD negative) and excellent responses in RAS, RUNX1 and IDH1/2 mutated AML cases. TP53-mutated patients responded poorly.

In the relapsed/refractory AML cohort of 32 patients, detailed responses by a molecularly-defined subgroup have been reported [91]. Patients with NPM1 mutations or mutations in the spliceosome genes, SRSF2 and ZRSR2, showed the highest response rates. Those patients with FLT3 or PTPN11 were less likely to respond, albeit with very small numbers. Data presented in abstract from at the 2018 ASH meeting would suggest patients with PTPN11 mutations display a unique metabolic profile and are sensitive to MCL-1 inhibitors in pre-clinical models [92]. A retrospective review of de novo and relapsed/refractory AML patients treated with venetoclax and HMAs was also reported in abstract form at the ASH 2018 meeting [93]. In multivariate analysis, only favourable or intermediate-risk cytogenetics were associated with better CR/Cri outcomes, with no difference across somatic mutations. In terms of overall survival using recursive partitioning analysis, mutations in SRSF2, IDH1/2 or RUNX1 were associated with improved outcomes, as were the presence of myeloid transcription factor or spliceosome-complex mutations. It is likely that further predictors of the response will be characterised over time with increasing numbers of patients treated with venetoclax, and that will aid in refining treatment approaches.

#### 3.6.2. BH3 Profiling

BCL-2 expression levels do not necessarily correlate with responses to venetoclax across a broad range of haematological neoplasms. Follicular Lymphoma, for example, ubiquitously expresses BCL-2 owing to the t(14;18) translocation which juxtaposes BCL-2 to the immunoglobulin heavy chain promotor; however, modest overall responses of 38% were observed in this disease in the Phase 1, first in human, trial of venetoclax [94]. In the same trial, only 18% of patients with diffuse large B cell lymphoma responded to venetoclax with no clear association between BCL-2 expression and response. It has emerged that the sensitivity of a cancer cell to BCL-2 inhibition depends, at least in part, due to the quantity of pro-apoptotic proteins that are bound by the BCL-2 molecule [95,96,97]. This so-called “priming” of BCL-2 and related anti-apoptotic family members can be measured using an in vitro technique known as BH3 profiling developed by Dr Letai and colleagues [98,99,100]. In short, the mitochondria of tumour cells are exposed to synthetic BH3 peptides that mimic the actions of pro-apoptotic BH3-only proteins, such as BIM. In doing so, the apoptotic threshold of the cancer cell can be determined. Further, using BH3 peptides with specificity for individual anti-apoptotic proteins, such as BCL-2, BCL-xL or MCL-1, the dependency of different cancers types on these various pro-apoptotic proteins can be determined. In the case of CLL, BH3 profiling at screening was shown to correlate with clinical outcomes [101]. Likewise, in AML, Konopleva et al. demonstrated that BH3 profiling could be used to predict responses to venetoclax monotherapy in relapsed/refractory patients [60]. Interestingly, BCL-2 dependency alone was not adequate to predict durable responses to venetoclax, but a lack of dependency of MCL-1 and BCL-xL was also required. It is yet to be determined if BH3 profiling can be used to improve clinical outcomes in patients by guiding treatment decisions with a future direction of clinical research.

## 4. Conclusions

Targeting BCL-2 is now considered a standard of care in the treatment of patients with chronic lymphocytic leukaemia and is emerging as a promising target in a number of haematological malignancies. Despite a dearth of new therapies over the last 30 years for patients with AML, the last few years have seen a relative explosion of FDA approvals, with the BCL-2 inhibitor venetoclax, being one of the most exciting. The responses seen in the Phase 2 trials combining venetoclax with both low dose cytarabine and hypomethylating agents in an upfront elderly patient cohort compare favourably to historical outcomes. We await the results of the Phase 3 trials to see if these results are confirmed. It must be noted, however, that venetoclax is no ’magic bullet’ in the treatment of AML. The median overall survival for patients treated with venetoclax plus hypomethylating agents was still only 15 months, which for an otherwise fit and healthy 75-year-old person, is a devastating outcome. In the high-risk subsets, including those with TP53 mutations and adverse risk cytogenetics, the outcomes are much worse than this, and highlight the ongoing unmet need for this group of patients with AML. In saying that, however, BCL-2 inhibition is only a very new strategy developed to target AML, and from the huge number of novel-novel combination trials ongoing, there is hope that both quality of life and survival outcomes can be further improved upon. Gaining a greater insight into predictors of both response and the biology of relapse post BCL-2 inhibitor therapy will help to grant finesse to treatment decisions. Targeting other anti-apoptotic proteins, such as MCL-1 and BCL-xL, in combination with venetoclax, seems an exciting prospect based upon pre-clinical work to date. After 30 years of “7+3,” there finally appears to be some new hope for the treatment of AML.

## Figures and Tables

**Table 1:** Ongoing clinical trials using BCL-2 Inhibitors in Acute Myeloid Leukaemia.

Clinicaltrials.gov Identifier	Target Group	Phase	Trial
NCT03484520	R/R AML (age ≥ 18)	I	A Study of Venetoclax and Dinaciclib (MK7965) in Patients With Relapsed/Refractory Acute Myeloid Leukemia
NCT03441555	R/R AML (age ≥ 18)	I	A Study of Venetoclax and Alvocidib in Patients With Relapsed/Refractory Acute Myeloid Leukemia
NCT02993523	AML (age ≥ 18; treatment-naïve)	III	A Study of Venetoclax in Combination With Azacitidine Versus Azacitidine in Treatment-Naïve Subjects With Acute Myeloid Leukemia Who Are Ineligible for Standard Induction Therapy
NCT03874052	R/R AML (age ≥ 18)	I	Ruxolitinib in Combination With Venetoclax for the Treatment of Relapsed/Refractory Acute Myeloid Leukemia
NCT03625505	R/R AML (age ≥ 18)	I	A Study to Assess Safety and Efficacy of Venetoclax in Combination With Gilteritinib in Subjects With Relapsed/Refractory Acute Myeloid Leukemia
NCT03573024	AML (ages 18–59; treatment-naïve)	II	Venetoclax and Azacitidine for Non-Elderly Adult Patients With Acute Myeloid Leukemia
NCT03069352	AML (age ≥ 18; treatment-naïve)	III	A Study of Venetoclax in Combination With Low Dose Cytarabine Versus Low Dose Cytarabine Alone in Treatment-Naive Patients With Acute Myeloid Leukemia Who Are Ineligible for Intensive Chemotherapy
NCT03672695	R/R AML (age ≥ 18)	I	Phase I Dose Escalation Study of Intravenously Administered S64315 (Mcl-1 Inhibitor) in Combination With Orally Administered Venetoclax in Patients With Acute Myeloid Leukaemia
NCT03844815	High risk AML (R/R AML, TP53 mutant, adverse cytogenetics; age ≥ 18)	I	Study of Venetoclax in Combination With Decitabine in Subjects With Acute Myeloid Leukemia
NCT03214562	Treatment naive and R/R AML (age ≥ 18)	I/II	Study of the BCL-2 Inhibitor Venetoclax in Combination With Standard Intensive Acute Myeloid Leukemia (AML) Induction/Consolidation Therapy With FLAG-IDA in Patients With Newly Diagnosed or Relapsed/Refractory Acute Myeloid Leukemia (AML)
NCT03709758	AML (age 18–75; treatment-naïve)	I	Venetoclax in Combination With Intensive Induction and Consolidation Chemotherapy in Treatment-Naïve AML
NCT03466294	AML (age ≥ 60; treatment-naïve)	II	Azacitidine and Venetoclax as Induction Therapy With Venetoclax Maintenance in the Elderly With AML
NCT03629171	R/R AML (age ≥ 18) with dose expansion cohort B for treatment-naïve	II	Liposome-encapsulated Daunorubicin-Cytarabine (CPX-351) and Venetoclax in Treating Participants With Relapsed, Refractory or Untreated Acute Myeloid Leukemia
NCT03455504	AML (age 18–65; treatment-naïve)	II	Venetoclax Add in Combination With Fludarabine, Cytarabine and Idarubicin in Induction for Acute Myeloid Leukemia (V-FIRST)
NCT03586609	AML (age ≥ 60; treatment-naïve)	II	Venetoclax, Cladribine, Low Dose Cytarabine, and Azacitidine in Treating Participants With Patients Previously Untreated Acute Myeloid Leukemia
NCT03735875	R/R AML (age 18–65)	I/II	Venetoclax and Quizartinib in Treating Patients With FLT3-mutated Recurrent or Refractory Acute Myeloid Leukaemia
NCT02670044	R/R AML (age ≥ 60)	I/II	A Study of Venetoclax in Combination With Cobimetinib and Venetoclax in Combination With Idasanutlin in Patients Aged >/= 60 Years With Relapsed or Refractory Acute Myeloid Leukemia Who Are Not Eligible for Cytotoxic Therapy
NCT03797261	R/R AML and R/R NHL/DLBCL (age ≥ 18)	I	A Study of Venetoclax and AMG 176 (MCL1 inhibitor) in Patients With Relapsed/Refractory Hematologic Malignancies
NCT03862157	Treatment naïve AML with a history of MDS, MPN, MDS/MPN, CNL, aCML, CEL (age ≥ 18)	I/II	Azacitidine, Venetoclax, and Pevonedistat in Treating Patients With Newly Diagnosed Acute Myeloid Leukemia
NCT03471260	R/R AML (age ≥ 18)	I/II	Study of Venetoclax With the mIDH1 Inhibitor Ivosidenib (AG120) in IDH1-Mutated Hematologic Malignancies
NCT03613532	High risk AML, MDS, MDS/MPN (age ≥ 18) going to Allogeneic SCT	I	Venetoclax Added to Fludarabine + Busulfan Prior to Transplant for AML, MDS, and MDS/MPN

Abbreviations: R/R, relapsed/refractory; AML, acute myeloid leukemia; MDS, myelodysplastic syndrome; MPN, myeloproliferative neoplasm; AUL, acute undifferentiated leukaemia; MPAL, mixed phenotypic acute leukaemia; CNL, chronic neutrophilic leukaemia; CEL, chronic eosinophilic leukaemia; aCML, atypical chronic myeloid leukaemia; NHL, non-Hodgkin lymphoma; DLBCL, diffuse large B cell lymphoma; SCT, stem cell transplant.

**Table 2:** Venetoclax Responses in Acute Myeloid Leukaemia.

Clinical Trial	Patients	Response	Response by Cytogenetic/Molecular Subtype
Venetoclax monotherapy in relapsed/refractory patients (NCT01994837)	*n* = 32	CR/CRi = 19% CR = 6% CRi = 13%	IDH1/2 = 33% CR/CRi SRSF2/ZRSR2 = 27% CR/CRi
Venetoclax + Hypomethylating Agents in treatment-naïve patients >65 years (NCT02203773)	*n* = 145	CR/CRi = 67% CR = 37% CRi = 30%	Poor Cytogenetics = 60% CR/CRi Intermediate Cytogenetics = 74% CR/CRi TP53 = 47% CR/CRi FLT3 = 72% CR/CRi IDH1/2 = 71% CR/CRi NPM1 = 91.5% CR/CRi
Venetoclax + low dose Cytarabine in treatment-naïve patients >60 years (NCT02287233)	*n* = 82	All patients CR/CRi = 54% CR = 26% CRi = 28% Without Prior HMA (N = 58) CR/CRi = 62% CR = 34% CRi = 28%	All patients Poor Cytogenetics = 42% CR/CRi Intermediate Cytogenetics = 63% CR/CRi TP53 = 30% CR/CRi FLT3 = 44% CR/CRi IDH1/2 = 72% CR/CRi NPM1 = 89% CR/CRi

Abbreviations: CR, complete remission; CRi, complete remission with incomplete haematological recovery; HMA, hypomethylating agent; IDH1/2, isocitrate dehydrogenase 1/2; TP53, tumour protein p53; FLT3, fms-like tyrosine kinase 3; NPM1, nucleophosmin 1.

## Abbreviations

AML	Acute Myeloid Leukaemia
MDS	Myelodysplastic Syndrome
7+3	7 days of cytarabine and days of an anthracycline
FLAG	Fludarabine, Cytarabine, Granulocyte Colony Stimulating Factor
FLAG-IDA	Fludarabine, Cytarabine, Granulocyte Colony Stimulating Factor and Idarubicin
HMA	Hypomethylating agent
LDAC	Low dose Ara-C
BCL-2	B cell lymphoma 2
BCL-xL	B cell lymphoma extra large
MCL-1	Myeloid Leukemia Cell Differentiation Protein
FLT3	Fms like tyrosine kinase 3
NPM1	Nucleophosmin
IDH1/2	Isocitrate Dehydrogenase 1/2
CR	Complete Remissiob
CRi	Complete remission with incomplete haematological recovery
PR	Partial Remission
R/R	Relapsed and Refractory
